# Burnout among staff on specialized eating disorder units in Norway

**DOI:** 10.1186/s40337-021-00473-x

**Published:** 2021-10-27

**Authors:** Trine Wiig Hage, Karin Isaksson Rø, Øyvind Rø

**Affiliations:** 1 Division of Mental Health and Addiction, Regional Department for Eating Disorders, Oslo University Hospital, Nydalen, P.O. Box 4956, 0424 Oslo, Norway; 2 LEFO - Institute for Studies of the Medical Profession, Oslo, Norway; 3 Department of Behavioral Medicine, Faculty of Medicine, University of Oslo, Oslo, Norway; 4 Faculty of Medicine, University of Oslo, Oslo, Norway

**Keywords:** Eating disorders, Burnout, Healthcare providers, Specialized eating disorder units, Work environment, Job satisfaction, Emotional dissonance

## Abstract

**Objective:**

Burnout is commonly associated with low workplace wellbeing. Patients with eating disorders are frequently referred to as a particularly challenging group to treat. It is therefore important to study healthcare providers´ workplace wellbeing in settings which treat eating disorders. The aims of the current study were to (a) measure burnout among healthcare providers working on specialized eating disorder units in Norway, and (b) explore factors predicting burnout.

**Methods:**

186 participants from 11 specialized eating disorder units in Norway completed an online survey including the Mashlach Burnout Inventory, and eating disorder-specific factors related to burnout*,* job satisfaction, work environment, emotional dissonance and stress. Multiple regression analysis was used to identify predictors of burnout.

**Results:**

Overall, low levels of burnout were found among the participants. Eating disorder-specific factors and emotional dissonance predicted the three central aspects of burnout, namely, emotional exhaustion, depersonalization, and a diminished sense of personal accomplishment.

**Conclusions:**

Findings suggest a relatively low level of burnout across age, gender, and professional categories working at specialized eating disorder units, contrary to commonly-held assumptions pertaining to the challenges involved in treating individuals with eating disorders.

## Plain English summary

Healthcare providers’ wellbeing at work is important for their health and their satisfaction and performance at work well as quality of patient care. Patients with eating disorders are frequently referred to as a particularly challenging group to treat, and may therefore affect healthcare providers’ working with this patient group sense of wellbeing. The current study thus aimed to measure levels of burnout and its relationship with associated aspects, e.g. work environment, job satisfaction, emotional dissonance and eating disorder specific factors among healthcare providers working specifically with this patient group. The outcome of the study showed relatively low levels of burnout among participating healthcare providers. Factors associated with eating disorders strongly predicted burnout.

## Background

Workplace wellbeing among healthcare providers is widely acknowledged as important for employees’ work performance and the quality of patient care (e.g. [1, 2]). One adverse consequence of low workplace wellbeing is burnout, which can be defined as “a psychological syndrome that involves a prolonged response to chronic interpersonal stressors on the job. The three key dimensions of this response are an overwhelming exhaustion, feelings of cynicism and detachment from the job and a sense of ineffectiveness and lack of accomplishment [3]. Healthcare providers who experience burnout are less likely to be responsive to patients’ needs and are more prone to leave their job, which can negatively affect the health and well-being of patients as well as the individual worker. Additionally, staff turnover can lead to a loss of organizational knowledge and available human resources in the healthcare sector, which may in turn have serious consequences on both an individual and societal level [4]. Studies from the medical field have shown a tendency towards a higher prevalence of burnout among doctors than nurses, measured by higher scores on one or more of the three dimensions in the self-report burnout questionnaires [5–7]. Other studies have not found such differences [8]. Furthermore, emotional dissonance, defined as a structural discrepancy between the real emotions experienced by the employee versus those expressed to conform to the work situation or job requirements [9], has been repeatedly linked with burnout among healthcare providers who perform emotionally challenging work (e.g. [10–12]).

Work-related factors commonly associated with burnout in the mental health care field include high work load, low experienced autonomy at work, role conflict, role ambiguity and work-related stress [13–15]. Regarding demographic predictors of burnout in the mental health field, existing research has produced inconsistent findings concerning age and gender [13, 15]. Few studies have examined differences in burnout among different professional groups within mental health. A recent study found that burnout associated with "interpersonal stress factors", i.e. work relationships (e.g. patients or relatives, colleagues and leaders) was higher among psychiatrists than among other hospital physicians [16]. The nature of mental health care, e.g. intense emotional involvement with patients over a prolonged period and exposure to traumatic content have been identified as possible contributors to burnout within this field [17]. Studies have also linked burnout among mental health care providers to various characteristics of the population served, e.g., in the addiction field [18]. Indeed, risk of burnout has been associated with treating complex disorders which are characterized by slow progress and susceptibility to relapse [15]. However, the majority of research on work-related wellbeing among healthcare providers assumes that findings are generalizable across settings and populations, which may conflate or overlook experiences of health‐care providers that are specific to the working context.

One area that has received insufficient attention in the literature involves burnout among healthcare providers treating patients with eating disorders (EDs). Patients with EDs have a reputation for being challenging to treat. Common explanations include the ego syntonic nature of the disorder, leading to patient ambivalence and resistance to treatment [19, 20]. In addition, there is a high risk of medical complications and death, particularly for patients with anorexia nervosa (AN) [21], as well as prolonged course and risk of relapse [22]. Negative reactions to patients with EDs, e.g. feeling frustrated, helpless or incompetent, among health care providers have previously been associated with higher levels of symptom severity, emotional dysregulation, and a diagnosis of AN, all of which are typical characteristics of patients admitted to specialized care [23]. However, a recent study found *high* job satisfaction among health care providers working on specialized ED units [24]. Additional research is necessary to investigate which specific aspects of working within specialized ED care are most challenging, and the resulting effect on burnout and wellbeing of healthcare providers employed in these settings.

Only a handful of studies have investigated burnout among healthcare providers working with patients with EDs. Consistently, studies found that the level of burnout was comparable to other populations within the mental health care field, with one study reporting moderate-to-high levels of emotional exhaustion in over half of the sample [25]. Younger, female professionals with less work experience were found to be at greater risk of burnout [25]. In terms of work-related predictors, a higher work load, organizational demands, and longer hours have been associated with burnout [25–27]. Additionally, ED- specific characteristics of the patient population, including high rates of relapse, the ego syntonic nature of the disorder, worry about patient death, and working with patients who are ambivalent or resistant to treatment were identified as a major source of burnout among participants [25–27]. These studies focused primarily upon individual therapists working in outpatient private practice [25, 27] or occupational therapists [26].There is a dearth of literature investigating burnout among healthcare providers with diverse professional backgrounds providing multidisciplinary treatment to the most severely ill patients within specialized treatment settings.

The aim of this study was to (a) measure burnout among healthcare providers working on specialized EDUs in Norway, and (b) investigate predictors of burnout (demographic, ED-specific, and work-related, i.e., job satisfaction, work environment, emotional dissonance and stress).

## Methods

### Design and setting

This cross-sectional study was part of a larger project investigating work-related well-being on specialized EDUs in Norway. Previously, we have examined job satisfaction among health care providers working on specialized units in Norway [24].

### Participants

The sample was comprised of healthcare personnel working on specialized EDUs in Norway. An invitation to participate and study information was sent via email to 11 specialized in- or outpatient units treating adults and adolescents with a primary diagnosis of an ED. 9 of the wards are organized within the Norwegian healthcare system and 2 of the wards are private institutions specialized in ED treatment. All units offer multidisciplinary treatment with an overarching aim of symptom reduction and somatic stabilization, in line with national guidelines [28]. On inpatient units, meal support and reduction of underweight are key therapeutic goals. A link to the online survey was provided. All permanent staff holding a minimum 50% clinical position was invited to participate. Participation was voluntary, and participants received no compensation. All individuals who agreed to participate were then asked to fill out a set of online questionnaires via Nettskjema, which is a tool for conducting online surveys operated by University of Oslo. Data were collected anonymously. Ethical approval was granted by the Norwegian Data Protection at Oslo University Hospital.

### Assessment

#### Overall burnout

The Mashlach Burnout Inventory (MBI) is a widely-used measure of job burnout [3]. For the present study, the 25-item Norwegian version of the MBI—Human Services Survey Medical Personnel (MBI-HSS) was used to measure burnout during the past two weeks. This version uses a five-point intensity scale (1-does not fit, 5-fits very well) and has shown to have satisfactory psychometric properties [29]. The MBI has 3 sub-scales: emotional exhaustion (MBI-EXH) (10 items), depersonalization/cynicism (MBI-DEP) (8 items) and reduced personal accomplishment (MBI-PA) (7 items) [29]. Similar to a prior study of a representative sample of Norwegian physicians, a cut-off of ≥ 3 scored on all three dimensions was used to define a “case” [30, 31]. In the present study, Cronbach’s alphas for the total and three subscales were 0.89, 0.92 for MBI-EXH, 0.63 for MBI-DEP, and 0.72 for MBI-PA.

#### Job satisfaction

The Job Satisfaction Scale (JSS) is a commonly-used questionnaire developed to measure job satisfaction [32]. The instrument is validated in Norwegian and has shown satisfactory psychometric properties [33]. The instrument consists of 10 items. Each item is scored on a seven-point Likert scale from 1 (very dissatisfied) to 7 (very satisfied). A total mean JSS score was calculated and the Cronbach’s alpha in the present study was 0.85.

#### Eating disorder-specific contributors to burnout

The EDBURN [25] is an 11-item questionnaire to evaluate factors conceptualized to contribute to burnout among ED treatment providers. The original 11-item questionnaire had a Cronbach’s alpha of 0.80 and was significantly correlated to the MBI subscales (r = 0.33 to r = 0.45). For the purpose of the current study, the following 4 items were used: treatment resistance/ego-syntonic nature, high risk of relapse, personality characteristics, and therapist’s level of concern about patient survival. Items were scored on a 5-point scale from 1 to 5 (1 = not at all to 5 = very much) and Cronbach’s alpha was 0.69.

#### Working environment

The General Nordic Questionnaire for Psychological and Social Factors at Work (QPS Nordic) has demonstrated psychometric properties across several organizations. For the purpose of the current study, the following 6 items were administered: “Do you perform job tasks for which you need more training?”, “Is your work challenging in a positive way?”, “Have you noticed any disturbing conflicts between co-workers?”, “Do you experience the climate in your work unit as safe and trust-based?”, “Is the relationship between you and your immediate superior a source of stress to you?” and “I am often discouraged at work and therefore often thinking about leaving my job.” Items are scored on a 5-point scale from 1 to 5 (1 = “very seldom or never” to 5 = “very often or always”). A total mean score was calculated and the Cronbach’s alpha for the 6 items was 0.75.

#### Psychosocial work characteristics

The Effort-Reward Imbalance Questionnaire (ERI) is a well-established questionnaire to assess psychosocial work characteristics, in particular, work-related stress [34]. For the present study, a 9-item version of the ERI was selected. The 9-item version consists of 4 items that comprise the effort scale (e.g. “Over the past few years, my job has become more and more demanding”) and 5 items which comprise the reward scale (e.g. “Considering all my efforts and achievements, my work prospects are adequate”). Participants indicate their response on a 5-point scale (1 = not demanding to 5 = very demanding) and an effort to reward ratio is calculated by dividing the effort score by the reward score. Cronbach’s alphas for the effort, reward, and total scale were 0.77, 0.74, and 0.79, respectively.

#### Emotional dissonance

The Frankfurt Emotional Work Scale was developed to assess appropriate regulation of emotions at work [9]. For the purpose of this study, the following 4 items were used: “How often in your job do you have to suppress emotions in order to appear neutral on the outside?” “How often in your job do you have to display emotions that do not agree with your true feelings?” How often in your job do you have to display pleasant emotions or unpleasant emotions on the outside while actually feeling indifferent inside?” and “How often in your job do you have to display emotions that do not agree with your actual feelings towards your clients?” Responses are scored on a scale from 1 to 5 (1 = seldom/never to 5 = several times an hour). In this study, Cronbach’s alpha was 0.85.

#### Demographic/individual difference and work-related variables

Participants answered questions regarding demographics (e.g., age, gender and profession), and work-related variables (e.g., work setting). We also included questions about self-reported work-related sickness absence and personal history of an ED.

### Statistical analysis

Means (SD) and the percentage of participants scoring above the pre-determined cut off score ≥ 3 on the MBI factors were reported. Student t-tests and ANOVA were carried out to test group differences. Multiple regression analysis was used to determine which variables of interest predicted MBI-EXH, MBI-PA and MBI-DEP: age, gender, profession, ED-specific contributors to burnout, job satisfaction, working environment, psychosocial work characteristics and emotional dissonance, and stress. Self-reported work related sickness absence and participants’ personal history of having an ED were not included due to a low number of participants endorsing these items. Forced entry method was selected and predictor variables were entered in 3 blocks: gender and age (block 1), gender, age and profession with nurses as the reference (block 2), and all variables (block 3). Alpha level was *p* < 0.05 and IBM SPSS statistics version 25 was used for the analyses.

## Results

A total of 285 healthcare providers were invited to participate in the study. Of these, 186 (65%) healthcare providers completed the online survey. The mean age was 44.5 years (range 24–66, SD = 11.7). In terms of gender, 170 (91.4%) of the participants were female and 16 (8.6%) were male. A total of 39 (21.0%) participants worked in an outpatient unit and 147 (79.0%) worked in an inpatient setting. Of 180 participants, 85 (47.2%) worked fixed daytime shifts and 95 (52.8%) worked rotating shifts or fixed nights shift. Six participants could not be classified into shift categories. The sample consisted of 93 (50.0%) nurses, 49 (26.3%) medical doctors and psychologists and 44 (23.7%) “other” professions, including social workers, physiotherapists and dieticians. The average duration of employment in the current position was 5.3 (SD 4.9) years. Only one participant (0.5%) had a current ED and 8 (4.3%) perceived themselves as recovered from an ED. There were 23 (12.3%) participants who reported self-reported sick leave during the past year, and 7 (3.7%) of the participants had been on sick leave the past month due to work-related factors.

Table 1 displays means (SD) for the MBI-EXH, MBI-PA, and MBI-DEP. A total of 15.6% scored above the cut-off (≥ 3) for emotional exhaustion (MBI-EXH), 8.6% for the lack of personal accomplishment (MBI-PA), and 0% for cynicism (MBI-DEP). No statistically significant differences in burnout were found for gender, treatment setting, or profession.

**Table 1:** Mean MBI (SD) subscale and percentage over cut-off score by demographic factors

		MBI-EXH	MBI-DEP	MBI-ACC
Total sample	N = 186	2.10 (0.72)	1.56 (0.39)	2.29 (0.45)
	N ≥ 3.0 (%)	29 (15.6%)	0 (0%)	16 (8.6%)
*Gender*				
Female	N = 170	2.13 (0.73)	1.57 (0.39)	2.30 (0.44)
	N ≥ 3.0 (%)	28 (17.5%)	0 (0%)	14 (8.2%)
Male	N = 16	1.88 (0.64)	1.53 (0.38)	2.22 (0.56)
	N ≥ 3.0 (%)	1 (6.3%)	0 (0%)	2 (12.5%)
*Age*				
< 45 years	N = 92	2.10 (0.74)	1.63 (0.42)*	2.25 (0.49)
	N ≥ 3.0 (%)	15 (16.3%)	0 (0%)	8 (8.7%)
≥ 45 years	N = 94	2.10 (0.71)	1.50 (0.35)*	2.33 (0.41)
	N ≥ 3.0 (%)	14 (14.9%)	0 (0%)	8 (8.5%%)
*Setting*				
Inpatient	N = 147	2.15 (0.73)	1.58 (0.41)	2.31 (0.45)
	N ≥ 3.0 (%)	24 (16.3%)	0 (0%)	13 (8.8%)
Outpatient	N = 39	1.91 (0.68)	1.51 (0.30)	2.25 (0.49)
	N ≥ 3.0 (%)	5 (12.8%)	0 (0%)	3 (7.7%)
*Day/shift*^†^				
Day workers	N = 85	2.09 (0.74)	1.56 (0.37)	2.27 (0.47)
	N ≥ 3.0 (%)	13 (13.3%)	0 (0%)	6 (7.1%)
Shift/night workers	N = 95	2.11 (0.71)	1.56 (0.41)	2.32 (0.44)
	N ≥ 3.0 (%)	14 (14.7%)	0 (0%)	10 (10.5%)
*Profession*				
Nurses	N = 93	2.01 (0.70)	1.55 (0.40)	2.32 (0.49)
	N ≥ 3.0 (%)	12 (12.9%)	0 (0%)	11 (11.8%)
Psychologists/medical doctors	N = 49	2.16 (0.71)	1.62 (0.38)	2.19 (0.45)
	N ≥ 3.0 (%)	9 (18.4%)	0 (0%)	2 (4.1%)
Others	N = 44	2.25 (0.77)	1.54 (0.40)	2.35 (0.35)
	N ≥ 3.0 (%)	8 (18.2%)	0 (0%)	3 (6.8%)

Bold is used to underline the most significant findings

MBI-EXH, emotional exhaustion; MBI-DP, cynicism; MBI-ACC, lack of personal accomplishment

^†^n = 6 not classified, *p < 0.05

There was a significant bivariate correlation (*p* < 0.001) between the majority of predictors and all three subscales of the MBI (Table 2). Age was significantly negatively correlated only to MBI-DEP (*p* < 0.05). The regression analyses showed that 63%, 33% and 22% of the variances of the final model explained MBI-EXH, MBI-DEP and lack of MBI-PA, respectively.

**Table 2:** Correlations between subscales of Mashlach Burnout Inventory and other variables

	MBI-EXH	MBI-DEP	MBI-ACC	JSS	EDB	ERI	FEWS	QPR
MBI_DEP	0.532***							
MBI_ACC	0.306***	0.254***						
JSS	− 0.473**	− 0.286***	− 0.285***					
EDB	0.511***	0.433***	0.321***	− 0.294***				
ERI	0.604***	0.318***	0.133	− 0.407***	0.336***			
FEWS	0.512***	0.455***	0.299***	− 0.412***	0.388***	0.337***		
QPS	0.690***	0.360***	0.297***	− 0.593***	0.358***	0.592***	0.457***	
Age	0.005	− 0.169*	0.081	− 0.052	0.003	0.119	− 0.104	− 0.029

MBI-EXH, emotional exhaustion; MBI-DP, cynicism; MBI-ACC, lack of personal accomplishment; JSS, job satisfaction scale; EDB, eating disorder-specific factors; ERI, psychosocial work characteristics; FEW, emotional dissonance; QPR, working environment

*p < 0.05, **p < 0.01, ***p < 0.001

ED-specific factors and emotional dissonance significantly predicted all three subscales of burnout, with the highest value for ED-specific factors (*p* < 0.05 to *p* < 0.001). Working environment and psychosocial work characteristics contributed significantly to the MBI-EXH (*p* < 0.001). Job satisfaction did not contribute to any of the models. Younger age predicted higher levels of cynicism as assessed by the MBI-DEP (*p* < 0.05) (see Table 3). The professions “psychologist/doctor” and “others” were significant predictors of MBI-EXH compared to nurses (*p* < 0.05).

**Table 3:** Forced entry regression predicting MBI subscale score

Predictor variables		Unstand. beta	Stand. beta	t-value	*p* value	R square
*Emotional exhaustion (MBI-EXH)*						
Gender	Block 1	0.25	0.10	1.33	0.18	
Age		0.00	0.01	0.17	0.87	**0.01**
Gender	Block 2	0.27	0.10	1.41	0.16	
Age		0.00	0.02	0.30	0.77	
Nurses versus psychologists/doctors		0.16	0.10	1.25	0.21	
Nurses versus other professions		0.25	0.15	1.87	0.06	**0.03**
Gender	Block 3	0.01	0.00	0.06	0.95	
Age		0.00	0.01	0.22	0.83	
Nurses versus psychologists/doctors		0.18	0.11	**2**.**16**	**0**.**03**	
Nurses versus other professions		0.19	0.11	**2**.**28**	**0**.**02**	
Job satisfaction scale (JSS)		− 0.04	− 0.05	− 0.75	0.45	
Eating disorder-specific factors (EDB)		0.27	0.23	**4**.**23**	**0**.**000**	
Emotional dissonance (FEW)		0.15	0.16	**2**.**96**	**0**.**003**	
Psychosocial work characteristics (ERI)		0.45	0.23	**3**.**88**	**0**.**000**	
Working environment (QPR)		0.44	0.37	**5**.**47**	**0**.**000**	**0.63**
*Cynicism (MBI-DP)*						
Gender	Block 1	0.02	0.01	0.17	0.86	
Age		− 0.01	− 0.17	− 2.30	0.02	**0.03**
Gender	Block 2	0.02	0.02	0.20	0.84	
Age		− 0.01	− 0.17	− 2.27	0.02	
Nurses versus psychologists/doctors		0.07	0.08	0.97	0.34	
Nurses versus other professions		− 0.02	− 0.02	− 0.27	0.79	**0.04**
Gender	Block 3	− 0.07	− 0.05	− 0.77	0.45	
Age		− 0.01	− 0.16	− **2**.**53**	**0**.**01**	
Nurses versus psychologists/doctors		0.06	0.07	0.94	0.35	
Nurses versus other professions		0.00	0.00	0.02	0.98	
Job satisfaction scale (JSS)		− 0.02	− 0.06	− 0.71	0.48	
Eating disorder-specific factors (EDB)		0.16	0.25	**3**.**51**	**0**.**001**	
Emotional dissonance (FEW)		0.13	0.25	**3**.**37**	**0**.**001**	
Psychosocial work characteristics (ERI)		0.13	0.12	1.47	0.14	
Working environment (QPR)		0.04	0.06	0.64	0.52	**0.33**
*Lack of personal accomplishment (MBI-ACC)*						
Gender	Block 1	0.09	0.06	0.75	0.45	
Age		0.00	0.09	1.16	0.25	**0.01**
Gender	Block 2	0.08	0.05	0.70	0.49	
Age		0.00	0.08	1.14	0.26	
Nurses versus psychologists/doctors		− 0.13	− 0.12	− 1.59	0.11	
Nurses versus other professions		0.04	0.04	0.46	0.65	**0.03**
Gender	Block 3	0.01	0.00	0.05	0.96	
Age		0.01	0.12	1.77	0.08	
Nurses versus psychologists/doctors		− 0.14	− 0.14	− 1.80	0.07	
Nurses versus other professions		0.08	0.08	1.06	0.29	
Job satisfaction scale (JSS)		− 0.02	− 0.04	− 0.47	0.64	
Eating disorder-specific factors (EDB)		0.20	0.27	**3**.**42**	**0**.**001**	
Emotional dissonance (FEW)		0.10	0.18	**2**.**19**	**0**.**03**	
Psychosocial work characteristics (ERI)		− 0.21	− 0.17	− 1.97	0.05	
Working environment (QPR)		0.13	0.17	1.77	0.08	**0.22**

Bold is used to underline the most significant findings

Given ED-specific factors were important predictors of all burnout subscales, a post hoc regression analyses was performed to test which of the ED-specific items contributed to burnout. The analyses showed that ED-specific contributors explained 26%, 22% and 12% of the variances of emotional exhaustion (MBI-EXH), cynicism (MBI-DEP) and lack of personal accomplishment (MBI-PA), respectively. The item “patient personality perceived to be difficult” was a highly significant predictor (*p* < 0.001) of all three subscales of burnout. The items “high relapse” and “therapist worry about patient’s survival” were significant predictors of EXH (*p* < 0.01 and *p* < 0.05, respectively) (Table 4).

**Table 4:** Forced entry of 4 items of Eating disorder-specific factors predicting MBI subscale score

Predictor variables	Unstand. beta	Stand. beta	t-value	*p* value
*Emotional exhaustion (MBI-EXH)* *R square = 0.26*				
Treatment resistance	0.06	0.07	0.82	0.42
High relapse	0.22	0.26	**3**.**15**	**0**.**002**
Patient personality perceived to be difficult	0.22	0.24	**3**.**36**	**0**.**001**
Therapist worry about somatic complications and patient’s survival	0.13	0.15	**2**.**21**	**0**.**028**
*Cynicism (MBI-DP)* *R square = 0.22*				
Treatment resistance	0.06	0.14	1.68	0.09
High relapse	0.05	0.11	1.29	0.20
Patient personality perceived to be difficult	0.18	0.35	**4**.**80**	**0**.**000**
Therapist worry about somatic complications and patient’s survival	0.00	0.00	-0.01	0.99
*Lack of personal accomplishment (MBI-ACC)* *R square = 0.12*				
Treatment resistance	0.03	0.07	0.73	0.46
High relapse	0.04	0.07	0.78	0.43
Patient personality perceived to be difficult	0.14	0.23	**2**.**98**	**0**.**003**
Therapist worry about somatic complications and patient’s survival	0.05	0.09	1.19	0.24

Bold is used to underline the most significant findings

## Discussion

Findings yield important information about demographic, eating disorder-specific, and work-related factors that contribute to burnout among healthcare providers working within specialized eating disorder care. Overall, findings indicated low levels of burnout among participants. Specifically, 16% of the sample scored above the cut-off on the emotional exhaustion subscale (MBI-EXH), which is considered the core symptom of burnout and most responsive to the psychosocial work environment [35]. No participants scored above cutoff on the depersonalization/cynicism subscale (MBI-DEP), and only 8.6% scored over cut-off score on the personal accomplishment scale (MBI-PA). In comparison, a study of 994 Norwegian physicians found that 29% scored above the cutoff on emotional exhaustion (MBI-EXH), 4% on the depersonalization/cynicism subscale (MBI-DEP) and 9% on personal accomplishment (MBI-PA) [29]. Our findings are in line with Warren et al. [25, 27], who found that participants working with patients with eating disorders were overall less burnt out compared to established norms for mental health care providers. One possible explanation for the relatively low levels of overall burnout in the current study is that although working with patients with eating disorders can be challenging, healthcare providers experience their work as important and engaging, an aspect that may have a protective effect against burnout [23, 25].

Several demographic variables, including being older and having more work experience, have previously been identified as protective factors against burnout among treatment providers working with patients with EDs [25]. In our study, however, none of the demographic variables (age, gender, professional category) significantly predicted burnout. Nevertheless, findings indicated an inverse relationship between age and personal accomplishment/cynicism (MBI-DEP), with younger age associated higher scores. This finding contrasts with results from a meta-analysis on burnout among mental health care providers, where increasing age was found to be associated with an increased risk of depersonalization [14]. Depersonalization, described by Maslach [3] as the act of distancing oneself from the patient, may be interpreted as a passive coping style. Moreover, due the vital role of therapeutic alliance within mental healthcare treatment, feeling distanced from the patients might have a greater negative impact on quality of patient care than within other medical settings [15]. More research is needed to further explore this relationship within an ED context.

We have previously found high job satisfaction in this sample [24]. The present data show that job satisfaction, as measured by the JSS, was also not associated with burnout. Previous research is inconclusive regarding the relationship between job satisfaction and burnout in mental health care [36, 37] as well as the general medical field [29]. However, findings from several studies indicate that job satisfaction can remain high despite burnout (e.g. [36, 38]).

Although not included in the study aims, we found one somewhat surprising and noteworthy finding in the data material. Only one participant reported having a current ED and few (4.3%) reported they were recovered from a prior ED. Compared to previous studies in the ED field, these numbers are surprisingly low. A higher lifetime prevalence of EDs among ED professionals has been found previously, approximately one-third (33.2%) in females [39]. Warren et al. [25] found that almost half of the sample reported having had an ED or high levels of ED symptoms in the past. Consequently, lived experience with EDs could be underreported in our sample. Differences between previous findings and the current study might be due to cultural differences across countries concerning the stigma of experiential knowledge within the ED treatment context. More research is warranted to explore this further.

Relative to nurses, belonging to the "psychologist/doctor" or "others" professional category significantly predicted the emotional exhaustion subscale (MBI-EXH) of burnout. Few studies, particularly within the mental health care field, have compared the prevalence of burnout between different professional groups. This is somewhat surprising, given that nurses, particularly nurses on inpatient units, spend more time with the patients than individual treatment providers. One possible explanation for this finding is that doctors and psychologists are in charge of the patient’s treatment plan on multiprofessional ED teams, and may be affected or burdened by this responsibility. Moreover, within the general medical field, higher levels of MBI-EXH among doctors than nurses are attributable to stressful working conditions, particularly a lower sense of job control [5], as well as differences in working conditions and managerial structure [7]. Findings from the current study point to the importance of tailoring interventions to reduce burnout among specific professional groups, and should be explored further in future studies. One example of such an intervention could be profession-specific supervision.

The third model including all predictors explained 63% of the total variance of MBI-EXH, which confirms that these variables together are important for understanding the emotional exhaustion dimension of burnout. Further, the QPS and ERI only predicted the MBI-EXH subscale, not the other dimensions of burnout. One possible explanation for this is that ERI, and partly the QPS, measure work-related stress, and thus, relates more to MBI-EXH than to the two other dimensions [40]. The variables predicted less of the total variance for depersonalization/cynicism (MBI-DP) and reduced personal accomplishment (MBI-PA), falling at 33% and 22%, respectively.

Of note, however, ED-specific factors significantly predicted all subscales on MBI. This finding is consistent with previous research highlighting that characteristics of this patient population are challenging to treat [25, 26]. Interestingly, the post hoc testing showed that the item “patient personality perceived to be difficult” was the strongest predictor of burnout among the items on EDBURN, and was significantly associated with all subscales of the MBI. This is in contrast to findings by Warren et al. [25], where the factor that contributed the most to perceptions of burnout was treatment resistance/ego-syntonic nature of EDs. One possible explanation for this discrepancy is that the healthcare providers in our study worked exclusively with EDs, mainly in inpatient and specialized settings, which typically treat the most severely ill patients. It is likely that these patients present with higher degrees of personality pathology [23], which is known to be associated with negative clinician reactions.

Emotional dissonance also predicted all three subscales of burnout. Expressing emotions of calmness, friendliness, and trustworthiness is often required when working with people with psychiatric disorders, and healthcare providers can thus be expected to act in particular ways even if they feel tired, upset or angry [9]. To our knowledge, no prior studies investigating the relationship between emotional dissonance and burnout have been conducted in the ED field, although a link between burnout and emotional dissonance has been established in other studies from the healthcare field [10]. This finding indicates that, even if caring for people in need can be experienced as a great source of motivation [41], emotional dissonance places the employee in a state of tension that can predict burnout. To witness the destructive eating behavior in patients with EDs is undoubtedly emotionally challenging for healthcare providers. Moreover, previous studies exploring treatment providers´ experiences working with patients with EDs have found that participants experience cognitive dissonance, i.e., mental discomfort that results from holding two conflicting beliefs, values, or attitudes, when treating these patients [19, 42]. The relationship between dissonance and the effect upon the wellbeing of healthcare providers as well as quality of patient care, should be explored in future studies.

## Limitations

Limitations should be acknowledged when considering the findings. The lack of a control group consisting of general mental health care providers limits the ability to discern whether findings are specific for ED treatment units in Norway, or applies generally to Norwegian health care providers. Moreover, due to the cross-sectional data of the study, findings cannot be used to infer cause and effect. Future research within this area should include a control group from other specialized units as well as from the generic psychiatric field. Given that this sample was self-selecting, it may be subject to selection bias. For instance, the sampling or assessment procedure may have excluded health care providers with high levels of burnout for various reasons (e.g., they were too overworked to complete the survey, were on long term sick leave or had already left the profession). Additionally, those who chose to participate in the study may have experienced less stress due to e.g. time constraints than the non-responders.

Several of the assessment measures were shortened versions that selected only a few items from the original scales. We also chose not to measure personality traits of the health care providers, although neuroticism has consistently been found to predict emotional exhaustion [40]. The use of single-item or abbreviated measures may affect validity. However, there are also potential advantages with using such measures, such as cost-efficiency, greater face validity, and the increased willingness of respondents to take the time to complete the questionnaire when the number of items is reduced.

## Conclusion

Overall, our findings suggest a relatively low level of burnout across age, gender, and professional categories working at specialized ED units, contrary to commonly-held assumptions pertaining to the challenges involved in treating individuals with EDs. However, ED-specific factors strongly predicted all dimensions of burnout (emotional exhaustion, depersonalization/cynicism, and reduced personal accomplishment). This underlines the importance of exploring specific factors related to the treatment population and setting when investigating burnout.

## Data Availability

The data that support the findings of this study are available upon request from the corresponding author.

## Abbreviations

ED: Eating disorder
AN: Anorexia nervosa
